# Splenectomy Fails to Provide Long-Term Protection Against Ischemic Stroke

**DOI:** 10.14336/AD.2018.0130

**Published:** 2018-06-01

**Authors:** Yuanyuan Ran, Zongjian Liu, Shuo Huang, Jiamei Shen, Fengwu Li, Wenxiu Zhang, Chen Chen, Xiaokun Geng, Zhili Ji, Huishan Du, Xiaoming Hu

**Affiliations:** ^1^China-America Institute of Neuroscience, Beijing Luhe Hospital, Capital Medical University, Beijing, China; ^2^Central Laboratory, Beijing Luhe Hospital, Capital Medical University, Beijing, China; ^3^Pittsburgh Institute of Brain Disorders and Recovery, and Department of Neurology, University of Pittsburgh School of Medicine, Pittsburgh, Pennsylvania 15213, USA

**Keywords:** cerebral ischemia, splenectomy, lymphocytes, neurological function

## Abstract

Splenectomy before or immediately after stroke provides early brain protection. This study aims to explore the effect of splenectomy on long-term neurological recovery after stroke, which is currently lacking in the field. Adult male rats were randomized into splenectomy or sham groups and then subjected to 90 min of middle cerebral artery occlusion (MCAO). Spleen was removed right upon reperfusion or 3d after MCAO. Infarct volume, neurological functions, and peripheral immune cell populations were assessed up to 28d after stroke. The results show that delayed removal of spleen did not reduce brain tissue loss and showed no effect on sensorimotor function (Rotarod, beam balance, forelimb placing, grid walk, and adhesive removal tests) or cognitive function (Morris water maze). Spleen removal immediately upon reperfusion, although significantly reduced the infarct size and immune cell infiltration 3d after MCAO, also failed to promote long-term recovery. Flow cytometry analysis demonstrated that immediate splenectomy after MCAO resulted in a prolonged decrease in the percentage of CD3^+^CD4^+^ and CD3^+^CD8^+^ T cells in total lymphocytes as compared to non-splenectomy MCAO rats. In contrast, the percentage of CD3^-^CD45RA^+^ B cells was significantly elevated after splenectomy. As a result, the ratio of T/B cells was significantly reduced in stroke rats with splenectomy. In conclusion, delayed splenectomy failed to provide long-term protection to the ischemic brain or improve functional recovery. The acute neuroprotective effect achieved by early splenectomy after stroke cannot last for long term. This loss of neuroprotection might be related to the prolonged disturbance in the T cell to B cell ratio.

Immune responses, which play a pivotal role in vascular aging and in the progress of brain injuries, are activated soon after ischemic stroke [1, 2]. As the largest lymphatic organ and a reservoir of immune cells, spleen has been incriminated for exacerbating acute brain injury at the early stage of stroke. Animal studies and clinical data consistently revealed a drastic shrinkage in spleen size in stroke victims, which is accompanied by the reduction in the number of splenic immune cells [3, 4]. Further studies demonstrated that both early splenectomy before middle cerebral artery occlusion (MCAO) and acute splenic irradiation after MCAO can alleviate the acute brain injury in animals [5, 6], indicating a detrimental role of splenic cells in acute phase of stroke. However, the function of spleen or the immune cells in spleen on long-term outcomes of stroke has not been explored.

Spleen contains a wide variety of immune cell populations, including lymphocytes, monocytes, neutrophils, and natural killer cells. Lymphocytes, including the B and T lymphocytes, are among the main residents in the spleen. Recent research in stroke field has highlighted the importance of lymphocytes in stroke outcomes. For example, the Rag1^-/-^ mice with T and B lymphocyte deficiency exhibited markedly reduced brain infarct and improved functional outcomes as compared to wild type controls, suggesting a harmful effect of the lymphocyte population in stroke [7, 8]. It is further noted that different lymphocyte subpopulations differentially contribute to the stroke pathology. While the T lymphocytes were shown to play a detrimental role in ischemia/reperfusion injury [7, 9], the B lymphocytes seemed to be protective after acute stroke [10]. Adding to this complexity, accumulating evidence also suggested the beneficial effects of regulatory immune cells, including regulatory T cells (Treg) [11-13] and regulatory B cells [10, 14, 15] in the ischemic brain. In contrast to the large amount of studies regarding the functions of lymphocytes in acute stroke, the influence of these cells in stroke recovery are still poorly understood. Investigating the function of spleen on long-term recovery after stroke remains an interesting topic, which will provide insights for further studies on the long-term effect of an individual immune cell population.

In the current study, we investigate the effects of spleen on long-term brain injury and neurological recovery after stroke using a 90 min MCAO rat model of stroke. Spleen was removed immediately upon reperfusion or 3d after MCAO to explore the long-term consequences in the presence or absence of early protection. We further explore the effects of splenectomy on the composition of peripheral immune cells after stroke. We found that delayed splenectomy failed to provide long-term protection to the ischemic brain or improve long-term functional recovery. The acute neuroprotective effect achieved by early splenectomy could not lead to long-term brain protection either. The prolonged change in the T cell to B cell ratio after spleen removal was observed in stroke rats, which might be related to the loss of protection in the long term.

## MATERIALS AND METHODS

### Animals

Male Sprague Dawley (SD) rats, weighing 280-320 g, were obtained from the Vital River Laboratory Animal Technology Co., Ltd. (Beijing, China). Animals were housed and given free access to food and water under 12 h light/dark cycle. All animal experiments were approved by the Animal Research Welfare Committee of Capital Medical University.

### Middle Cerebral Artery Occlusion Model of Stroke

Transient focal cerebral ischemia was induced in male rats by 90 min occlusion of right middle cerebral artery under isoflurane inhaled anesthesia as described previously [6]. The body temperature was controlled at 37.0 ± 0.5°C throughout the surgery using a heating pad. The cerebral blood flow was measured by Laser Doppler flowmetry. The MCAO was carried out by inserting the nylon thread tip (2838-A1, Beijing Cinontech Co. Ltd., China) into the internal carotid artery. The surgical suture was removed and allowed for reperfusion after 90 min of occlusion. Rats were survived for 3, 5, or 28 days following initiation of reperfusion. Animals that died or failed to show at least 70% regional CBF reduction of the pre-ischemia levels were excluded from further analyses. All rats were randomly assigned into 4 groups using a lottery drawing box: Sham, Sham + Splenectomy, MCAO, MCAO + Splenectomy. Sham-operated animals were subjected to a surgery to separate the right carotid artery shorn of occlusion underwent the same anesthesia. A total of 136 rats were used, including 11 rats that were excluded from further assessments due to death after surgery. The mortality rate was 0% in sham group (0/6), 0% in sham + splenectomy group (0/6), 9.10% in MCAO group (6/66) and 8.62% in MCAO + splenectomy group (5/58).

### Splenectomy

Upon immediate reperfusion or 3 days after perfusion in stroke rats, both Sham + Splenectomy and MCAO + Splenectomy rats were anesthetized with a mixture of isoflurane (1.5% to 2.0%) and oxygen. Approximately 1cm of incision through a midline laparotomy was open to pull out the spleen. The splenic vessels were cauterized and removed [16].

### Analysis of cell populations by flow cytometry

Rats were anesthetized. Blood samples (500 μl) were collected by cardiac puncture and put into a 2 ml centrifuge tube with anticoagulants. Red cell lysis buffer (Beyotime Biotechnology Co. Ltd., Jiangsu, China) were used following manufacturer’s instructions to remove red blood cells. The cells were stained by different antibodies for flow cytometry analysis (Cytomics FC 500, Beckman coulter), including CD4^+^ T cells (CD3^+^CD4^+^), CD8^+^ T cells (CD3^+^CD8^+^), B lymphocytes (CD3^+^CD45RA^+^), NK cells (CD3^-^CD161a^+^), and monocytes (CD3^-^CD43^+^). For brain cell collection, rats were anesthetized and transcardially perfused with 0.9% NaCl. The ischemic hemisphere was dissected. The cells were isolated from brain tissues for flow analysis on a BD flow cytometer using FACS Diva 6.0 software. The data were analyzed by FlowJo (BD, USA).

### Acute infarct volume quantification with 2, 3, 5-Triphenyltetrazolium Chloride (TTC) Staining

For TTC (Sigma-Aldrich) staining, brains were harvested on day 3 after reperfusion in immediate splenectomy experiment, and 5 days after reperfusion in delayed splenectomy experiment. 2-mm-thick coronal sections were cut and stained with 2% TTC at 37°C, as previously described [17]. Infarct volume (cortex, striatum, and hemisphere), was analyzed using the image analysis software (Image J) and was expressed as the volume of the contralateral region minus the non-infarcted volume of the ipsilateral region.

### Neurological deficit assessments

Neurological deficits were assessed before surgery and 0.5h, 24h, 48h, and 72h after brain reperfusion by an investigator blinded to experimental groups. Longa scoring system [18] (0 = no deficit, 1 = failure to extend left forepaw, 2 = circling to the left, 3 = falling to the left, 4 = failure to walk spontaneously and loss of consciousness, 5 = death) was used.

### Neurobehavioral Tests

To assess whether delayed splenectomy treatment would improve motor functions, five sensorimotor behavioral tests were conducted at 3, 7, 14, 23, and 28 days following reperfusion. These tests included rotarod, adhesive removal, beam balance, forelimb placing, and grid walk test. Cognitive function test (Morris water maze) was performed at 23-28 days after reperfusion.

### Rotarod test

The rotarod test was performed as previously described [19]. Briefly, rats were placed on a rotating drum, and forced to run with speed accelerating from 4 to 40 rpm within 300s. The time until the animals dropped the rotating rod was measured. Rats were tested 5 times daily with an intermission of 5 minutes.

### Adhesive removal

The motor impairments of the forepaw were analyzed by adhesive tape removal test, which is a bilateral asymmetry paw test as previously described [20]. Adhesive tape was attached on the palmar surface of the forepaw. The time taken for the first attempt to touch and the time taken to remove the tape were recorded within a maximum of 120 seconds. The tests were performed 3 times on the day prior to the surgery and each day after MCAO. Data of the contralateral forepaw (impaired) were analyzed.

### Beam balance test

The motor coordination was evaluated by beam walk test as previously described [21]. Rats were individually handled with care on a narrow wooden beam (122 cm long, 2.5 cm wide, and 42 cm height), and pre-tested to pass through the beam voluntarily without a slip. The motor performance was scored from 0 to 6 as follows: 0 = no attempt to stay on beam; 1 = attempts to stay on beam but no movement; 2 = attempts to cross the beam but fail; 3 = cross the beam but the contralateral hindlimb slips >50%; 5 = cross the beam but the contralateral hindlimb slips <50%; 6 = cross the beam without a slip.

### Forelimb-placing test

Forelimb-placing test was used to assess the response of rats to sense tactile stimulation from vibrissa and visual [22]. In this test, rats were held gently with forelimbs closing to the tabletop and lightly brushed the tabletop with each side of their vibrissa. The examiner recorded the ability of rats to place the preferred forelimb on the edge of the table. Each forelimb of rats was repeatedly tested for 10 trials, and the placing rate (/10) was calculated.

### Grid walk test

The ability of rats accurately placing the forepaws during spontaneous walking was evaluated by grid walk test [23]. Rats were placed on the wire grid (100 cm long, 25 cm wide, and 50 cm height with 35×35mm grid squares) and allowed to freely walk from one end to the other. The total number of foot slips was recorded when the forepaw failed to accurately hold on the rung.

### Morris Water Maze

Morris water maze test was used to assess the long-term cognitive function. In brief, a circular platform (diameter 10cm) was submerged in one quadrant for 1 cm of circular pool (diameter 150 cm) containing opaque water at 25 °C. The pool was divided into four quadrants by phantom lines. To evaluate the learning ability, rats were placed into the pool from one of the four locations and allowed to swim for 90 s to find the hidden platform. After each trial, rats were placed on the platform for 30 s and memorized the site with marked spatial cues around the pool. Swim speed and the latency for rats to find the hidden platform were recorded at 24-28 days after MCAO.

### Immunofluorescence staining

Rats were sacrificed under anesthesia at 28 days after MCAO. The brains were perfused with 0.9% saline followed by 4% paraformaldehyde in phosphate buffer (PBS, pH 7.4). The brains were harvested, fixed with 4% paraformaldehyde for 48 hours, and sank in serial sucrose solutions (20% and 30%). Frozen brain tissues were cut into 30μm sections by a cryotome. All samples were attached to slides and stored in slide box at -20 °C.

For immunohistochemistry staining, sections were washed in PBST (3×10min, pH 7.4), and incubated with 10% serum for 30 min at room temperature. The sections were then incubated with primary antibody (rabbit anti-MAP-2 antibody, 1:400, AB5622, Millipore) overnight at 4°C. Sections were washed with PBST (3×10min) and incubated with secondary antibodies (conjugated with Alexa Fluor 488 goat anti-rabbit IgG, 1:100, A11034, Life Technologies; Alexa Fluor 488 donkey anti-mouse IgG, 1:100, A21202, LifeTechnologies) for 2 h at room temperature. After the final wash with PBST (3×10min), sections were counterstained with the nuclear staining (DAPI fluoromount-G, 0100-20, Southern Biotech) and covered with coverslips. All samples were examined under a laser scanning confocal microscope.

### Statistical Analysis

Data are presented as mean ± standard error of mean (SEM). Multiple comparisons were performed using a one-way or two-way ANOVA followed by the Bonferroni post hoc test. Comparisons of two groups were done by the Student’s t-test. Statistical analyses were performed using GraphPad Prism 5. P values less than 0.05 was considered to be statistically significant.

**Figure 1.**
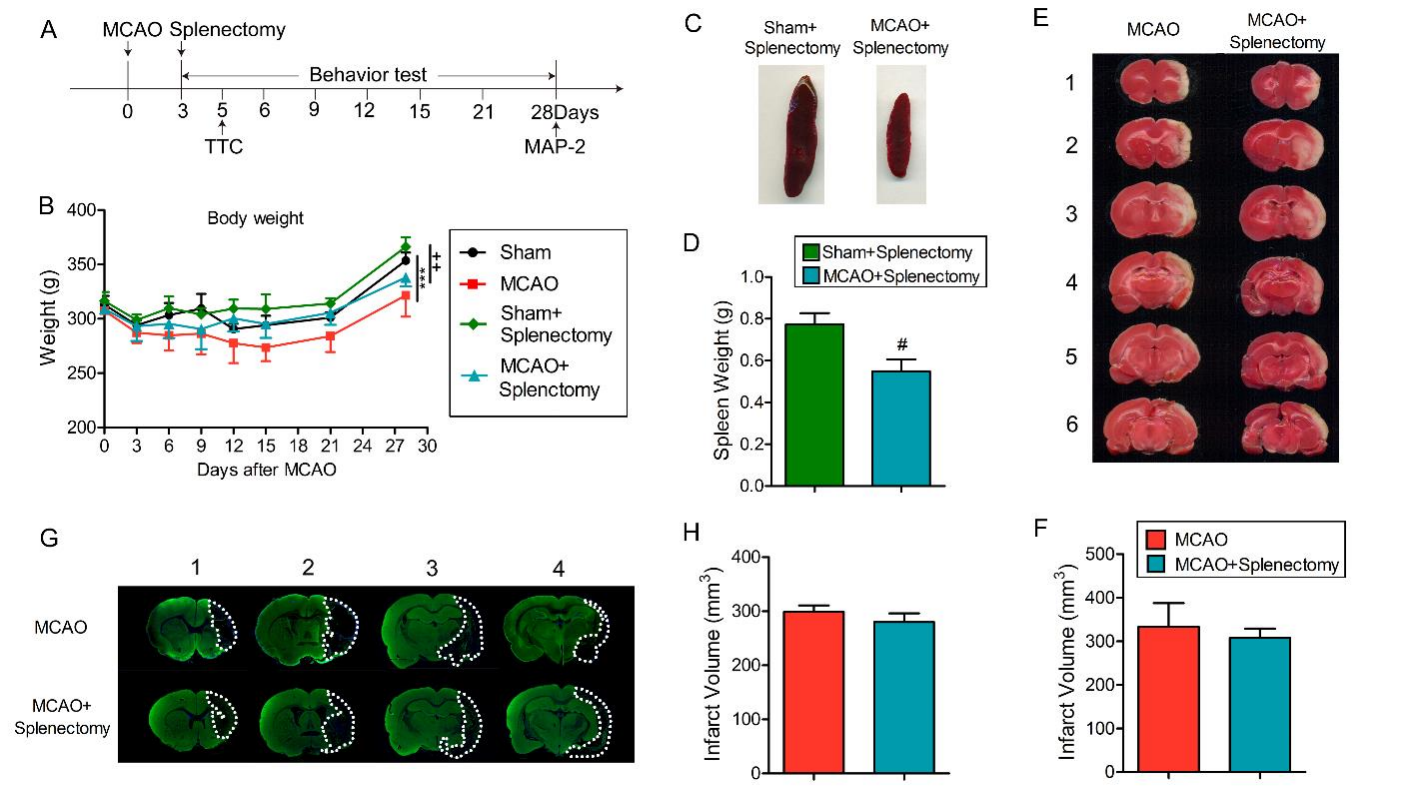
Delayed splenectomy after ischemic stroke shows no effect on acute and long-term brain tissue loss. Rats were subjected to 90 min MCAO followed by delayed splenectomy at 3 days after ischemic stroke. (A) Illustration of the experimental timelines. (B) Body weight was examined up to 28 days after MCAO. n=6 rats for sham groups; n=9 rats for MCAO groups. ***p<0.001: Sham vs MCAO; ^+++^p<0.001: Sham+Splenectomy vs MCAO+Splenectomy; ^##^p<0.01: MCAO vs MCAO+Splenectomy group by one-way ANOVA repeated measurement. (C) Representative images of spleen from sham+splenectomy and MCAO+splenectomy groups at 3 days after MCAO. (D) Quantification of spleen weight at 3 days after stroke. n=9 for each group. #p<0.05 by Student’s *t*-test. (E) Representative images of TTC-stained coronal brain sections from MCAO and MCAO + splenectomy groups at 5 days after MCAO. (F) Quantification of infract volume 5 days after MCAO. n=5-6 for each group. #p<0.05 by Student’s *t*-test. (G) Representative images of MAP-2 staining in MCAO and MCAO + splenectomy groups at 28 days after MCAO. (H) Quantification of brain tissue loss at 28 days after MCAO. n=5-6 for each group. Values are mean ± SEM.

**Figure 2.**
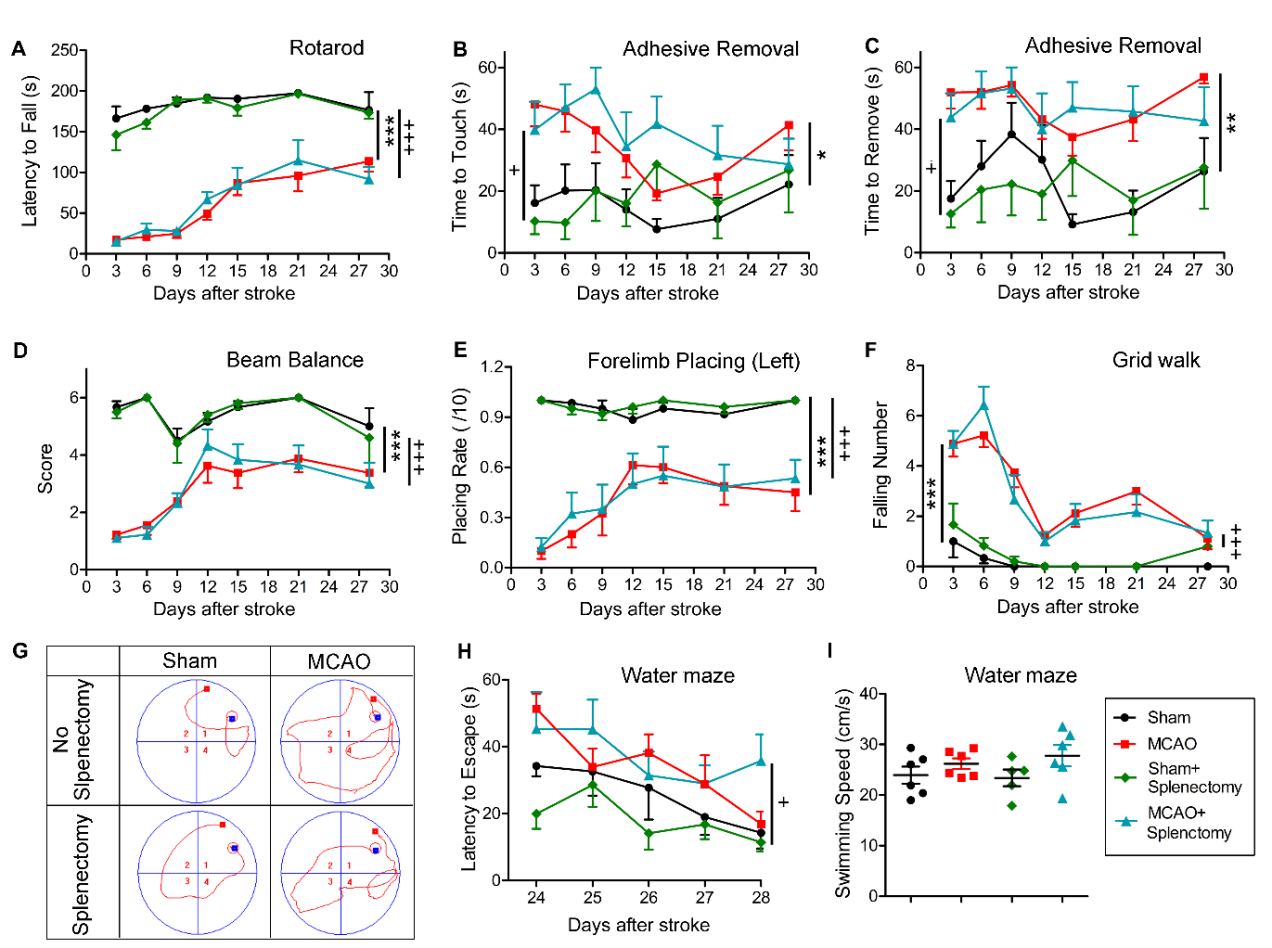
Delayed splenectomy after ischemic stroke failed to improve long-term neurological functions. Rats were exposed to 90 min of MCAO, followed by splenectomy or mock surgery right upon reperfusion as described in Materials and Methods. Sham-operated rats with or without splenectomy were used as control. All rats were allowed to survive for 28 days. (A) Rotarod test. The latency to fall off Rotarod was recoded. (B-C) The adhesive removal test. The time to touch (B) and the time to remove the tapes (C) were recorded. (D) Beam balance test. The performance on beam was scored 1-6. (E) The forelimb placing test on left (lesion side) forelimb. The number of successful placing out of 10 trials was recorded. (F) The grid walk test was assessed by counting the number of forelimb falling. (A-F) n=6 rats for sham groups; n=9 rats for MCAO groups. (G-I) Learning ability was examined by the Morris water maze test at 24-29 days after MCAO. n=5-6 rats per group. (G) Representative images of the swim paths at 28 days after MCAO (H) Latency to locate the submerged platform at 23-28 days after MCAO. (I) Swim speed at 28 days after MCAO. Values are mean ± SEM. Sham vs MCAO group: *p ≤ 0.05, **p ≤ 0.01, ***p ≤ 0.001 by two-way ANOVA repeated measurements. Sham+Splenectomy vs MCAO+Splenectomy group: ^+^p ≤ 0.05, ^+++^p ≤ 0.001 by two-way ANOVA repeated measurements.

## RESULTS

### Delayed splenectomy cannot protect against MCAO-induced acute and long-term brain injury

To investigate the effect of delayed splenectomy after stroke, we removed the spleen at a relatively late time points (3d) after stroke (Fig. 1A). The MCAO rats, either with or without splenectomy, experienced more body weight loss following reperfusion compared to the corresponding sham group (p<0.001 for non-splenectomy; p<0.01 for splenectomy by one-way ANOVA repeated measurement) (Fig. 1B). Consistent with previous reports [24], the size and weight of spleen in MCAO rats were much smaller compared to sham rats (Fig. 1C-D, p<0.05 by Student’s *t* test). Delayed splenectomy showed no effect on infarct volume at early (5d, Fig. 1E-F, p>0.05 by Student’s *t* test) or late stage (28d, Fig. 1G-H, p>0.05 by Student’s *t* test) after MCAO. These data demonstrated that delayed splenectomy resulted in comparable infarct volume in splenectomy and non-splenectomy rats after MCAO. Since it excludes the difference in early infarction, the delayed splenectomy could be a good model to evaluate the effect of spleen on long-term functional outcomes after stroke.

**Figure 3.**
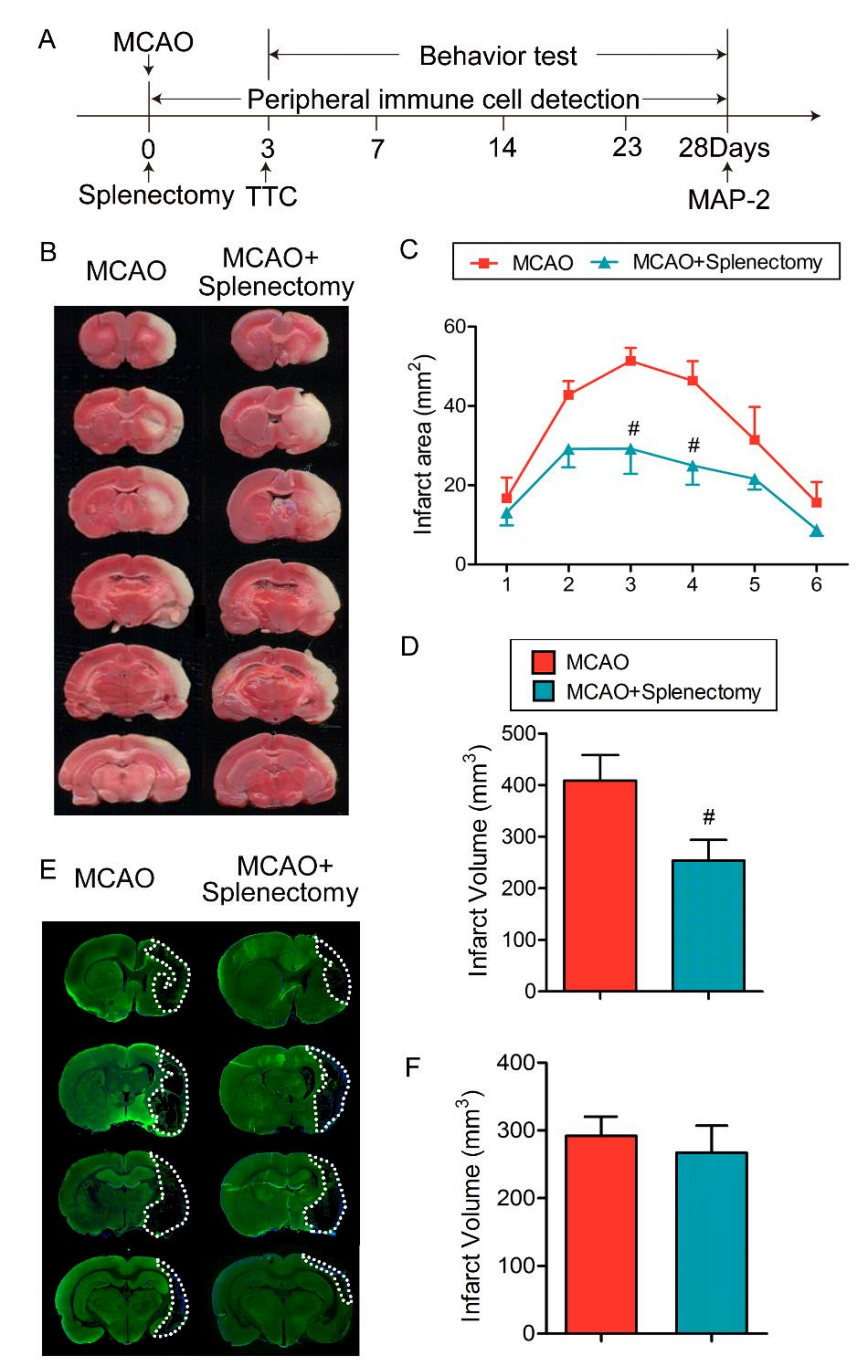
Splenectomy immediately after ischemic stroke reduces brain infarct early after MCAO but shows no effect on long-term brain damage. Rats were subjected to 90 min of MCAO followed by immediate splenectomy. (A) Illustration of the experimental timelines. (B) Representative images of TTC-stained coronal brain sections at 3 days after 90 min MCAO. (C) Quantification of infarct areas in each slice. n=5-6 rats per group. #p<0.05 by two-way ANOVA. (D) Quantification of infract volume. n=5-6 rats per group. #p<0.05 by Student’s *t*-test. (E) Representative images of MAP-2-stained coronal brain sections at 28 days after MCAO. (F) Quantification of infarct volume at 28 days after MCAO. n=5-6 rats per group. Values are mean ± SEM.

### Delayed splenectomy fails to improve long-term functional recovery after MCAO

We then determined the effect of delayed splenectomy on long-term neurofunctional performance after ischemic stroke. Sensorimotor functions were evaluated by Rotarod, adhesive removal, beam balance, forelimb placing, and grid walk tests. As shown in Figure 2A, the latency to fall off the Rotarod was significantly reduced in MCAO rats as compared to sham-operated rats (p<0.001, sham vs MCAO by one-way ANOVA repeated measurement). Splenectomy had no influence on Rotarod performance in sham-operated animals (p>0.05, sham vs sham + splenectomy by one-way ANOVA repeated measurement). Rats subjected to MCAO followed by delayed splenectomy demonstrated impaired behavioral performance as compared to sham animals with delayed splenectomy (p<0.001, sham + splenectomy vs MCAO + splenectomy by one-way ANOVA repeated measurement). Importantly, delayed splenectomy did not change the latency to fall off Rotarod in MCAO rats (p>0.05, MCAO vs MCAO + splenectomy by one-way ANOVA repeated measurement). Similar results were observed in adhesive removal test (Fig. 2B-2C), beam balance test (Fig. 2D), forelimb placing test (Figure 2E), and Grid walk test (Fig. 2F). Delayed splenectomy had no effect on sensorimotor functions in sham-operated rats or stroke rats.

We also evaluated the cognitive deficits 24-28 days after stroke using Morris water maze (Fig. 2G-2I). The latency for the rats to find the submerged platform was recorded (Fig. 2H). The MCAO rats with delayed splenectomy or mock surgery showed comparably elongated latency to locate the hidden platform as compared to their respective sham controls (Fig. 2H, p<0.05, sham vs MCAO; p<0.01, MCAO vs MCAO + splenectomy; p>0.05, MCAO vs MCAO +s plenectomy by one-way ANOVA repeated measurement). All groups showed no significant difference in swim speed at 28 days after MCAO or sham operation (Fig. 2I). These data suggest that delayed splenectomy did not result in significant improvement in spatial learning capacity after stroke.

**Figure 4.**
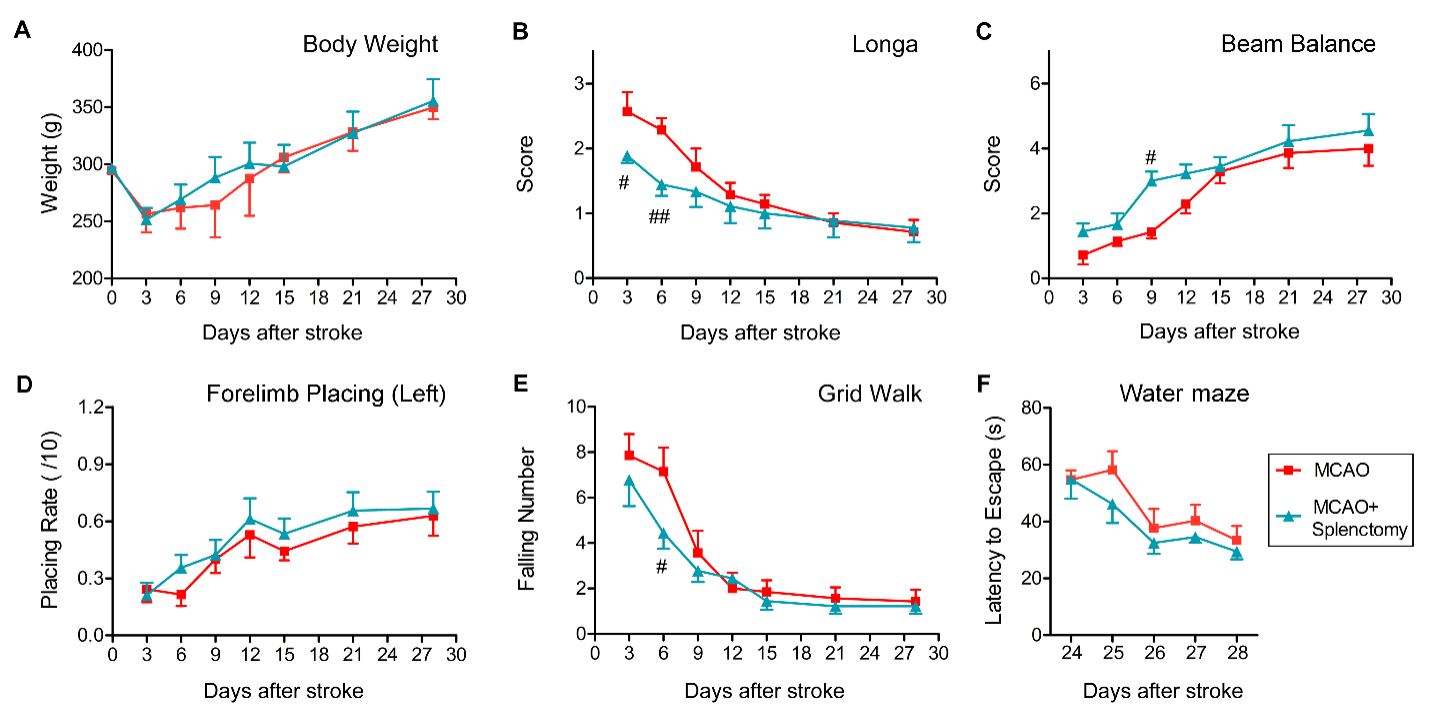
Splenectomy immediately after ischemic stroke cannot improve long-term functional recovery after MCAO. Rats were subjected to 90 min of MCAO followed by immediate splenectomy. (A) Body weight was recorded up to 28 days after MCAO. (B) The longa score was examined. (C) Beam balance test. The performance on beam was scored 1-6. (D) The forelimb placing test on left (lesion side) forelimb. The number of successful placing out of 10 trials was recorded. (E) The grid walk test was assessed by counting the number of forelimb falling. n=7 for MCAO groups; n=9 for MCAO+Splenectomy groups. (F) Cognitive ability was revealed by the Morris water maze test at 24-29 days after MCAO. n=6 rats per group. Values are mean ± SEM. ^#^p ≤ 0.05, ^##^p ≤ 0.01 by two-way ANOVA repeated measurements.

### Splenectomy immediately after ischemic stroke reduces brain infarct early after MCAO but shows no effect on long-term brain damage

Splenectomy before or immediately after ischemic stroke was reported to reduce brain infarct in acute stages after stroke [6, 25]. Here, we investigated whether neuroprotection provided by early splenectomy could last for long term (Fig. 3A). Our result shows that although significantly reduced brain infarct at 3 days after tMCAO (Fig. 3B-3D, p<0.05 by two-way ANOVA in C and Student’s *t* test in D), early splenectomy right after MCAO failed to reduce brain tissue loss at 28 days after MCAO (Fig. 3E-3F, p>0.05 by Student’s *t* test). These results confirmed that splenectomy could not provide long-term protection to the ischemic brain.

We then investigated the effect of acute splenectomy on long-term neurofunctional performance after ischemic stroke. Acute splenectomy had no influence on body weight in MCAO rats (Fig. 4A, p>0.05, by two-way ANOVA repeated measurement). The score of longa was significantly reduced on days 3 and 6 after stroke in MCAO + Splenectomy rats as compared to MCAO rats but showed no improvement at later time points (Fig. 4B, 3d p<0.05, 6d p<0.01, by two-way ANOVA repeated measurement). Rats subjected to MCAO followed by acute splenectomy have increased beam balance score only on day 9 after stroke as compared to MCAO animals (Fig. 4C, 9d p<0.05, by two-way ANOVA repeated measurement). Acute splenectomy had no effect on forelimb placing test (Fig. 4D), grid walk test (Fig. 4E) or water maze test (Fig. 4F) after stroke. These data suggested that acute splenectomy might result in a transient improvement in functional performance; however, it could not promote long-term functional recovery after MCAO.

**Figure 5.**
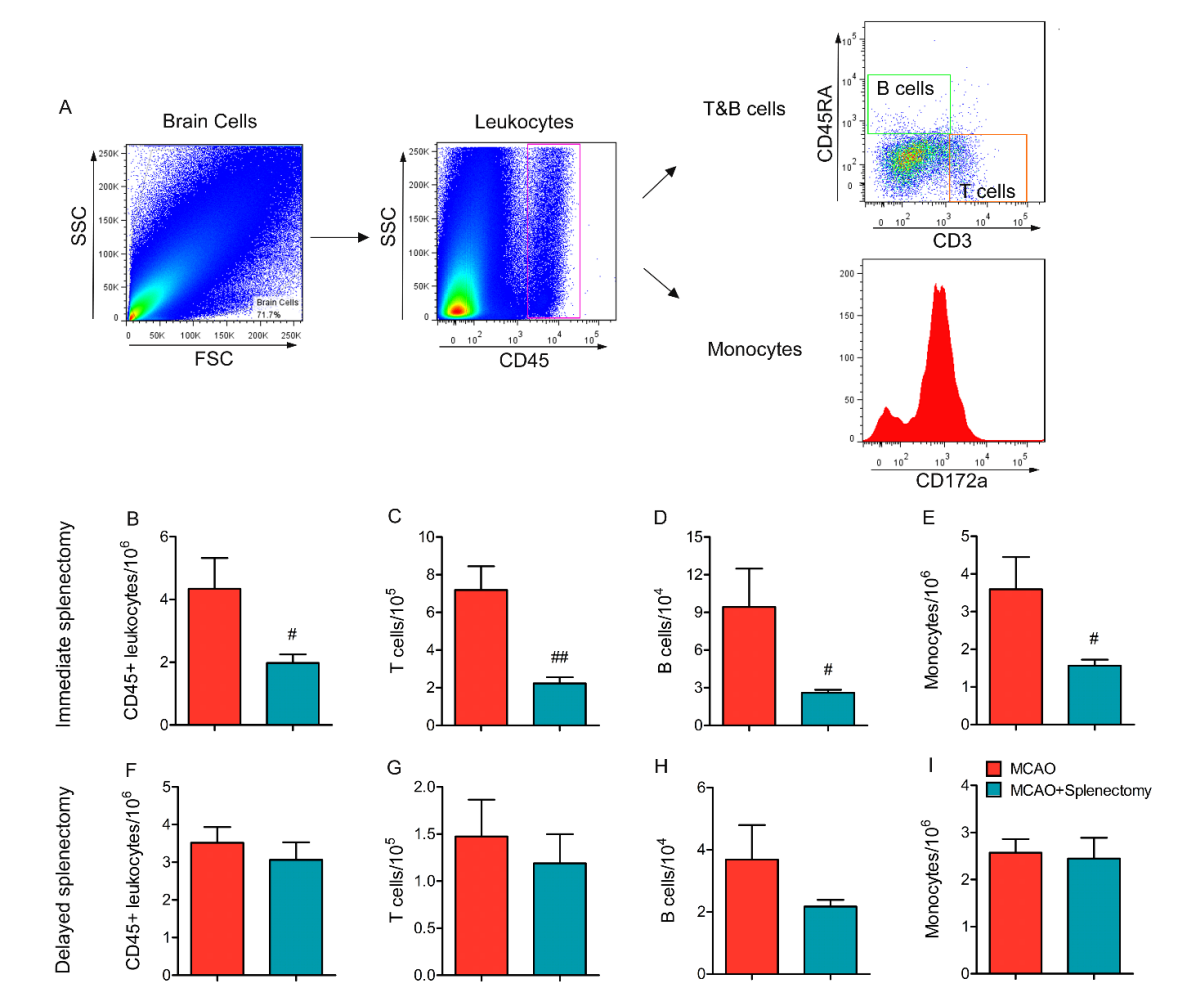
Immediate splenectomy, but not delayed splenectomy, reduces peripheral immune cell infiltration into the ischemic brain. Rats were subjected to 90 min MCAO followed by immediate splenectomy or delayed splenectomy (3d after MCAO) as described in Materials and Methods. (A) Representative gating strategy for CD45^+^ leukocytes, CD45^+^CD3^+^CD45RA^-^ T cells, CD45^+^CD3^-^CD45RA^+^ B cells, and CD45^+^CD43^+^ monocytes. (B-E) Flow cytometry analysis on the number of immune cell infiltration at 3 days after MCAO and immediate splenectomy. (B) Quantification of the number of CD45^+^ leukocytes. (C) Quantification of the number of CD45^+^CD3^+^CD45RA^-^ T cells. (D) Quantification of the number of CD45^+^CD3^-^CD45RA^+^ B cells. (E) Quantification of the number of CD45^+^CD43+ monocytes. (F-I) Flow cytometry analysis on the number of immune cell infiltration in rats with or without delayed splenectomy at 5 days after MCAO. (F) Quantification of the number of CD45^+^ leukocytes. (G) Quantification of the number of CD45^+^CD3^+^CD45RA^-^ T cells. (H) Quantification of the number of CD45^+^CD3^-^CD45RA^+^ B cells. (I) Quantification of the number of CD45^+^CD43^+^ monocytes. n=6 rats per group. Values are mean ± SEM. ^#^p<0.05, ^##^p<0.01 by 2-tailed Student’s *t* test.

**Figure 6.**
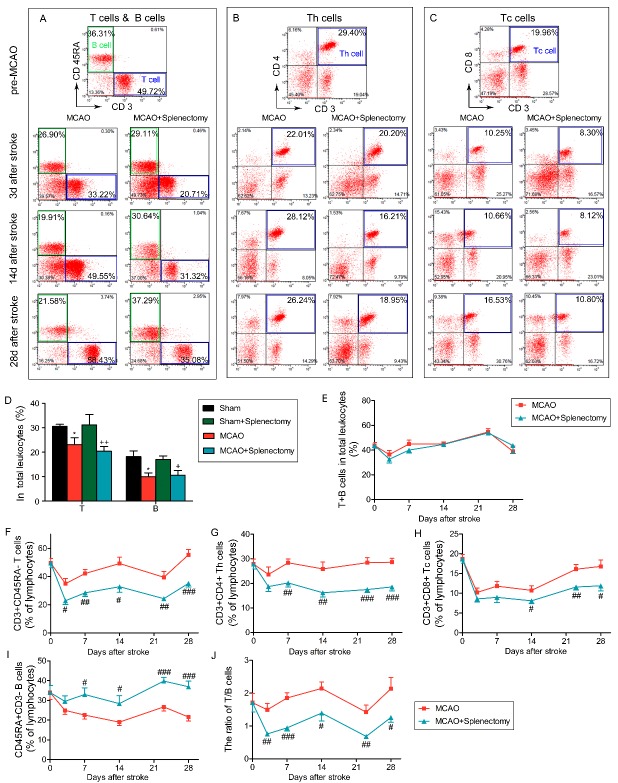
Splenectomy results in elevated ratio of B cells in lymphocytes for at least 28 days after MCAO. Rats were subjected to 90 min of MCAO followed by immediate splenectomy. (A-C) Representative flow cytometry plots of CD3^+^CD45RA^-^ T cells (A), CD3^-^CD45RA^+^ B cells (A), CD3^+^CD4^+^ Th cells (B), CD3^+^CD8^+^ Tc cells (C) in total lymphocyte in MCAO and MCAO+splenectomy groups at pre-MCAO, 3, 14 and 28 days after MCAO. (D) The percentage of T and B cells among leukocytes at 3 days after MCAO. n=9-12 rats per group. *p<0.05: Sham vs MCAO; +p<0.05, ++p<0.01: Sham+splenectomy vs MCAO + splenectomy by one-way ANOVA. (E) The percentage of T+B cells in total leukocytes in MCAO and MCAO + splenectomy groups over time. (F-J) The percentage of CD3^+^CD45RA^-^ T cells (F), CD3^+^CD4^+^ Th cells (G), CD3^+^CD8^+^ Tc (H), CD3^-^CD45RA^+^ B cells (I) in lymphocytes in MCAO and MCAO+splenectomy groups over time. (J) The ratio of T/B cells in MCAO and MCAO+splenectomy groups over time. Values are mean ± SEM. ^#^p<0.05, ^##^p<0.01, ^###^p<0.001 by two-way ANOVA. n=9-12 rats per group.

### Immediate splenectomy, but not delayed splenectomy, reduces peripheral immune cell infiltration early after stroke

To determine the impact of immediate and delayed splenectomy on peripheral cell infiltration, we prepared single cell suspension from ischemic hemispheres of MCAO and MCAO+Splenectomy rats after stroke (Fig. 5). Immediate splenectomy significantly reduced the number of CD45^+^ infiltrated leukocytes at 3d after MCAO (Fig. 5B, p<0.05 by Student’s *t* test). Specifically, the numbers of infiltrated CD45^+^CD3^+^CD45RA^-^ T cells, CD45^+^CD3^-^CD45RA^+^ B cells and CD45^+^CD43^+^ monocytes all significantly reduced in MCAO rats with immediate splenectomy as compared to MCAO rats without splenectomy (Fig. 5C-5E, p<0.05 by Student’s *t* test). In contrast, delayed splenectomy failed to diminish leukocyte infiltration into the ischemic brain at 5d after stroke (Fig. 5F-5I, p>0.05 by Student’s *t* test).

**Figure 7.**
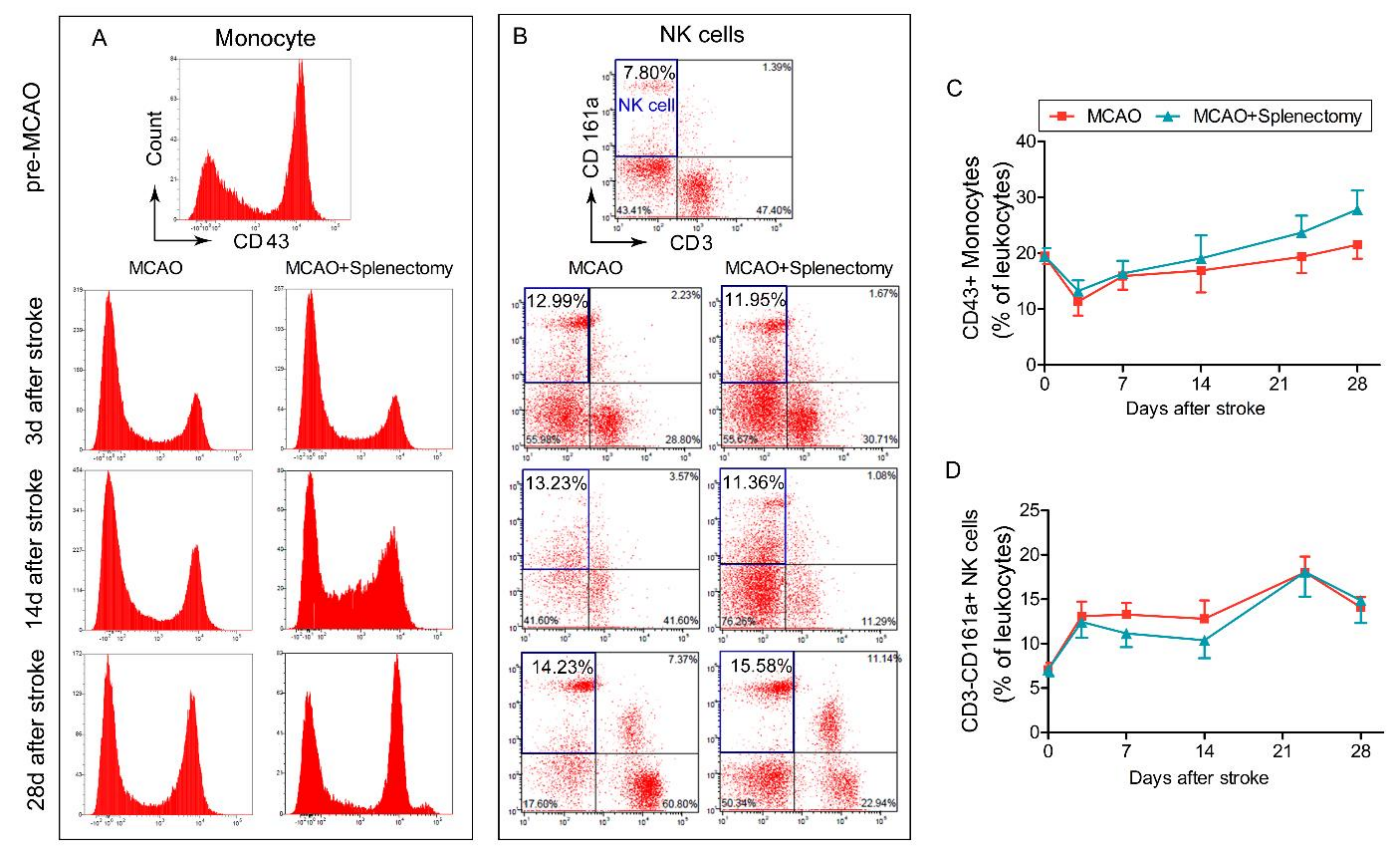
Splenectomy shows no effect on percentage of monocytes or NK cells for at least 28 days after MCAO. Rats were subjected to 90 min of MCAO followed by immediate splenectomy. (A-B) Representative flow cytometry plots of CD43^+^ monocytes (A) and CD3^-^CD161a^+^ NK cells (B) in total leukocytes in MCAO and MCAO+splenectomy groups at pre-MCAO, 3, 14, and 28 days after MCAO. (C) The percentage of CD43^+^ monocytes in total leukocytes in MCAO and MCAO+splenectomy groups over time. (D) The percentage of CD3^-^CD161a^+^ monocytes in total leukocytes in MCAO and MCAO+splenectomy groups over time. n=9-12 rats per group.

### Splenectomy results in elevated ratio of B cells in lymphocytes for at least 28 days after MCAO

Spleen is one of the main repertoires for immune cells, especially lymphocytes. We therefore evaluated the effect of splenectomy on the composition of immune cells after stroke. As shown in Figure 6A, splenectomy itself did not cause changes in the frequency of T cells or B cells in total leukocytes. MCAO led to significant decreases of the percentage of T cells and B cells in the blood at 3 days after ischemic stroke with or without splenectomy (Fig. 6D, T cells: MCAO vs sham p<0.05, MCAO + splenectomy vs sham + splenectomy p<0.01; B cells: MCAO vs sham p<0.05, MCAO+splenectomy vs sham+splenectomy p<0.05 by one-way ANOVA). Further analysis showed that the total lymphocyte percentage (T+B, Fig. 6E, p>0.05 by two-way ANOVA) remained the same between MCAO and MCAO + splenectomy group. However, splenectomy resulted in changes in the composition of lymphocytes. The percentage of CD3^+^CD45RA^-^ T cells (Fig. 6A and 6F, p<0.05 by two-way ANOVA), including CD3^+^CD4^+^ Th cells (Fig. 6B and 6G, p<0.05 by two-way ANOVA) and CD3^+^CD8^+^ Tc cells (Fig. 6C and H, p<0.05 by two-way ANOVA) among total lymphocytes after stroke significantly decreased in rats with splenectomy. In contrast, the percentage of CD3^-^CD45RA^+^ B cells (Fig. 6A and 6I, p<0.05 by two-way ANOVA) among total lymphocytes after stroke significantly increased in rats with splenectomy. As a result, there was a significant decrease in the ratio of T/B cells in MCAO+splenectomy on days 3 (p<0.01), 7 (p<0.01), 14 (p<0.05), 21 (p<0.01) and 28 (p<0.05) after stroke (Fig. 6J, two-way ANOVA).

MCAO led to no significant changes in the percentage of CD3^-^CD43^+^ monocytes (Fig. 7A and 7C, p>0.05 by two-way ANOVA) and CD3^-^CD161a^+^ NK cells (Fig. 7B and 7D, p>0.05 by two-way ANOVA) in the blood for at least 28 days after ischemic stroke with or without splenectomy. It indicated that splenectomy shows no effect on the percentage of circulating monocytes or NK cells for at least 28 days after MCAO.

Taken together, our results demonstrated a prolonged change in the lymphocyte composition, with an increase in B cell population, in stroke rats with spleen removal.

## DISCUSSION

The present study explored the effect of splenectomy on long-term outcomes after ischemic stroke. Spleen is the largest secondary lymphoid organ that serves as a storage site for immune cells, as well as a site of hematopoiesis. The movement of spleen-released immune cells into the blood and their subsequent infiltration into the ischemic brain is thought to exacerbate brain damage during acute stages of stroke. Although gender difference may exist [25], majority of animal studies in rodents agree that the inhibition of splenic function prior to or immediately after MCAO is neuroprotective and significantly reduces cerebral inflammation early after stroke [16]. However, whether and how the spleen responses contribute to long-term outcomes after stroke is not clear. Our study fills this gap by showing the effect of splenectomy on structural and functional deficits up to 28 days after ischemic brain injury. In our first experiment, we removed the spleen 3 days after stroke. Such delayed splenectomy had no early protection and no effect on immune cell infiltration early after stroke, and therefore excluded the possible long-term effects that are secondary to the early changes in infarct volume. Our results suggest that such delayed splenectomy had no impact on long-term brain tissue loss or functional outcomes after stroke. Interestingly, even when spleen was removed immediately after stroke, the reduction in brain infarct could only be observed at early stage after ischemia, which is accompanied by reduced immune cell infiltration. In other words, inhibition of peripheral immune responses by immediate spleen removal can provide a transient protection to the ischemic brain but has no beneficial effects on long-term protection. Taken together, these results strongly suggest that spleen removal cannot provide protection to the ischemic brain in the long run. In consistent with our conclusion, a recent study observed the lack of effect of splenectomy on long-term sensory motor functions after stroke [26].

The importance of immune cells on the pathology of ischemic stroke has been widely accepted. We therefore characterized the immune cell composition in the blood of immediately splenectomized stroke rats in order to dissect the possible mechanism for the loss of long-term protection. Our data suggested that splenectomy resulted in a selective and prolonged increase in B cell ratio in lymphocytes and a simultaneously decrease in the frequencies of T cells, including CD4^+^ Th cells and CD8^+^ Tc cells after stroke. In line with our results, the elevation of B lymphocytes in the blood has been previously reported in splenectomized rats, which were attributed, at least partially to the low recirculating efficiency of B cell population compared to T cells, through lymph nodes [27, 28]. Similarly, the splenectomized patients had markedly elevated numbers of immunoglobulin-secreting B cells in circulation [24]. Such changes in lymphocyte composition after splenectomy are particularly interesting, given previous data obtained in rodents that demonstrated the dependence of functional outcomes after stroke on the immune cell composition, especially the lymphocyte populations after ischemic stroke [29]. Actually, the lack of effect of splenectomy on long-term outcomes after stroke could possibly be explained by the diversity of lymphocytes in the spleen and the distinct roles of different lymphocyte populations under pathological conditions. In the context of ischemic stroke, it is known that the activation of CD8^+^ cytotoxic T cells contributes to long-term neurological deficits [30]. B-lymphocyte-mediated autoimmune response has also been shown to be related to delayed cognitive dysfunction after brain ischemia [20, 31]. The reduction of CD8^+^ cytotoxic T cells in splenectomized rats is thus expected to improve stroke outcomes. However, the elevation in B cell population may result in deteriorated long-term deficits after stroke. Therefore, spleen removal-resulted elevation in B lymphocytes and reduction in CD8+ T cells may counteract each other and lead to minimal changes in final stroke outcomes. In addition, recent studies have shown that several specific lymphocyte subpopulations, such as regulatory T lymphocytes [11, 13, 32, 33], influence long-term recovery after stroke. With the development of new techniques for cell tracking [34], the contribution of these specific lymphocytes in spleen to long-term stroke outcomes could be further investigated.

The monocyte-derived macrophages, although not involved in acute infarct development, [35] have been shown to assume different phenotypes after stroke and play an important role for stroke recovery [21, 22]. In addition, it is reported that splenectomy results in neuroinflammation and glial activation [36]. However, the relationship between splenectomy and microglia/macrophage activation in stroke model is unclear. Nevertheless, our data suggests reductions in the number of infiltrated lymphocytes and monocytes after immediate splenectomy. All these cells, especially T cells and monocytes, may influence microglia/macrophage activities in the ischemic brain. Further analysis on the microglia/monocyte/macrophage phenotype is warranted to fully understand the potential impact of splenectomy on microglia/macrophages after MCAO and the role of these cells on long-term recovery after stroke.

In summary, our study demonstrated that delayed splenectomy failed to provide long-term protection to the ischemic brain or improve long-term functional recovery. The acute neuroprotective effect achieved by early splenectomy immediately after stroke cannot last for long-term either. The prolonged disturbance in the B cell and T cell composition after spleen removal might be related to the lack of protection in long-term run. Further experiments on specific cell depletion and adoptive transfer are warranted to confirm the function of each spleen-derived cell population in long-term recovery after stroke and identify the cellular targets for restorative treatment.
